# Bat Rabies and Human Postexposure Prophylaxis, New York, USA

**DOI:** 10.3201/eid1712.102024

**Published:** 2011-12

**Authors:** Millicent Eidson, Yoichiro Hagiwara, Robert J. Rudd, Louise-Anne McNutt

**Affiliations:** New York State Department of Health, Albany, New York, USA (M. Eidson, Y. Hagiwara, R.J. Rudd);; University at Albany School of Public Health, Rensselaer, New York, USA (M. Eidson, Y. Hagiwara, L.-A. McNutt)

**Keywords:** viruses, zoonoses, rabies, bats, human, public health, postexposure prophylaxis

**To the Editor:** The New York State Department of Health (NYSDOH) assessed the effect of terrestrial rabies on human postexposure prophylaxis (PEP) during the first 10-year period of computerized reporting (1993–2002) (*1*). We assessed the effect of bat rabies during the same period, when guidelines for PEP were changing (*2*). NYSDOH developed local health department and public education programs to reduce bat encounters, increase testing of bats involved in encounters, and improve reporting of bat encounters (*3*).

Use of PEP for all New York counties was included in the study; PEP in New York, New York, and from other states was excluded. Analyses of reasonable probability exposures, age, and sex were conducted for 1998–2002. Population data from 2000 (www.factfinder.census.gov) were used to calculate rates. Epi Info (Centers for Disease Control and Prevention, Atlanta, GA, USA) and SAS (SAS Institute, Cary, NC, USA) were used for χ^2^ statistical analyses. We considered p values <0.05 significant.

During 1993–2002, a total of 6,320 bat-associated rabies exposure incidents and 11,365 PEPs were reported (Table). Incidents increased 7-fold, and use of PEP increased 9-fold. More than three quarters of all incidents were reported in June, July, and August. The number of persons who received PEP per incident ranged from 1 to 40, with an increase in mean from 1.3 to 1.8.

**Table:** Bat-associated rabies exposure incidents, PEP, and bats received for testing, New York, USA, 1993–2002*

Incidence data	No. (%)

1993	1994	1995	1996	1997	1998	1999	2000	2001	2002	Total
Total incidents†	137 (100.0)	116 (100.0)	290 (100.0)	527 (100.0)	672 (100.0)	764 (100.0)	964 (100.0)	924 (100.0)	973 (100.0)	953 (100.0)	6,320 (100.0)
Bats tested†	42 (30.7)	43 (37.1)	57 (19.7)	111 (21.1)	111 (16.5)	116 (15.2)	112 (11.6)	110 (11.9)	113 (11.6)	124 (13.0)	939 (14.9)
Bats not tested	95 (69.3)	73 (62.9)	233 (80.3)	416 (78.9)	561 (83.5)	648 (84.8)	852 (88.4)	814 (88.1)	860 (88.4)	829 (87.0)	5,381 (85.1)
Total PEP	184	131	440	968	1,326	1,512	1,755	1,641	1,735	1,673	11,365
Average/incident	1.3	1.1	1.5	1.8	2.0	2.0	1.8	1.8	1.8	1.8	1.8
Bat rabies status											
Positive†	49 (26.6)	17 (13.0)	34 (7.7)	74 (7.6)	88 (6.6)	111 (7.3)	110 (6.3)	110 (6.7)	99 (5.7)	98 (5.9)	790 (7.0)
Negative	6 (3.3)	24 (18.3)	17 (3.9)	37 (3.8)	36 (2.7)	33 (2.2)	22 (1.3)	32 (2.0)	21 (1.2)	23 (1.4)	251 (2.2)
Untestable	18 (9.8)	10 (7.6)	34 (7.7)	110 (11.4)	89 (6.7)	69 (4.6)	74 (4.2)	76 (4.6)	114 (6.6)	115 (6.9)	709 (6.2)
Not tested	111 (60.3)	80 (61.1)	355 (80.7)	747 (77.2)	1,113 (83.9)	1,299 (85.9)	1,549 (88.3)	1,423 (86.7)	1,501 (86.5)	1,437 (85.9)	9,615 (84.6)
Bat exposure type											
Bite†	43 (23.4)	71 (54.2)	124 (28.2)	160 (16.5)	188 (14.2)	134 (8.9)	186 (10.6)	163 (9.9)	150 (8.6)	145 (8.7)	1,364 (12.0)
Scratch or saliva contact	73 (39.7)	50 (38.2)	102 (23.2)	259 (26.8)	429 (32.4)	168 (11.1)	147 (8.4)	131 (8.0)	152 (8.8)	126 (7.5)	1,637 (14.4)
Reasonable probability	NA	NA	NA	NA	NA	1,145 (75.7)	1,365 (77.8)	1,299 (79.2)	1,382 (79.7)	1,367 (81.7)	6,558 (57.7)
Other	68 (37.0)	10 (7.6)	214 (48.6)	549 (56.7)	709 (53.5)	65 (4.3)	57 (3.2)	48 (2.9)	51 (2.9)	35 (2.1)	1,806 (15.9)
Bats received for rabies testing											
Total	420 (100.0)	419 (100.0)	386 (100.0)	764 (100.0)	741 (100.0)	868 (100.0)	923 (100.0)	1,220 (100.0)	1,421 (100.0)	1,487 (100.0)	8,649 (100.0)
By bat rabies status											
Positive‡	20 (4.8)	17 (4.1)	19 (4.9)	23 (3.0)	28 (3.8)	38 (4.4)	34 (3.7)	36 (3.0)	45 (3.2)	34 (2.3)	294 (3.4)
Negative	342 (81.4)	375 (89.5)	315 (81.6)	653 (85.5)	667 (90.0)	769 (88.6)	833 (90.2)	1,112 (91.1)	1,300 (91.5)	1,363 (91.7)	7,729 (89.4)
Untestable	58 (13.8)	27 (6.4)	52 (13.5)	88 (11.5)	46 (6.2)	61 (7.0)	56 (6.1)	72 (5.9)	76 (5.3)	90 (6.1)	626 (7.2)
By exposure type											
Bite†	77 (18.3)	106 (25.3)	103 (26.7)	118 (15.4)	98 (13.2)	139 (16.0)	141 (15.3)	131 (10.7)	131 (9.2)	148 (10.0)	1,192 (13.8)
Nonbite§	343 (81.7)	313 (74.7)	283 (73.3)	646 (84.6)	643 (86.8)	729 (84.0)	782 (84.7)	1,089 (89.3)	1,290 (90.8)	1,339 (90.0)	7,457 (86.2)

*PEP, human rabies postexposure prophylaxis; NA, data not collected for this time period. †Test for trend, p<0.0001. ‡Test for trend, p<0.005. §Includes scratch, saliva, and reasonable probability.

Nonbite exposures (scratch, direct and indirect contact with saliva, reasonable probability of exposure, and other unspecified exposures) accounted for 88% of PEP, with a significant increasing trend. During 1998–2002, “reasonable probability” and “bat in the bedroom” accounted for 79% and 53% of bat-associated PEP, respectively.

Rabies-positive bats accounted for 7% of PEP, with a significant decreasing trend. Untested bats accounted for 89% of the increase in PEP. Three quarters of PEP was administered for nonbite exposures to untested bats.

Of 8,244 PEPs since 1998, a total of 4,384 (53.2%) were for female patients, for whom the age-adjusted rate was 15.6 PEPs per 100,000 persons per year, compared with 14.3 for male patients (p = 0.0003). Persons <14 years of age received PEP twice as often as did persons >15 years of age. More persons <14 years of age (86%) received PEP for reasonable probability of exposure than did persons >15 years of age (76%) (p = 0.001).

During the study period, a total of 8,649 bats were received for rabies testing with concerns reported at the time of submission about the possibility of human contact, although further epidemiologic review would not classify them all as exposure incidents (Table). The number of bats submitted increased almost 4-fold. Similar to the seasonal pattern of exposure incidents, three quarters of bats were received for testing during June through August, with most (40%) received during August. Three percent of submitted bats were rabies positive, 89% were rabies negative, and 7% were unsatisfactory for testing. There was a significant decreasing trend in the proportion of tested bats that were rabid.

Bats for which nonbite contacts were reported accounted for 86% of those received for testing and 93% of the increase in bats received. There was a significant increasing trend in the proportion of bats reported with nonbite contacts.

For bats not tested, encounters resulted in an average of 1.8 PEP per incident, at an estimated cost for biologics of $10.9 million based on an average of $1,136 per PEP (*4*). Capturing and testing the 7,729 rabies-negative bats precluded the need for ≈14,000 PEP at an estimated savings for biologics of $15.8 million.

Encounters with bats are fairly common in New York State. Eidson et al. reported that one-third of survey respondents reported a bat in their house, including 10% who had seen a bat in their bedroom (*3*). Less than 20% knew a bat found indoors should not be released until rabies exposure is ruled out.

Similar rabies patterns have been reported from other states and Canada. In Massachusetts the number of bats submitted for rabies testing increased substantially during 1985–2009 (*5*). South Carolina reported an increase in administration of bat-associated PEP during the same period as this study (*6*). The seasonal pattern of bat encounters in New York was similar to those reported in Colorado (*7*), Minnesota (*8*), and Quebec, Canada (*9*), reflecting the pattern of bat hibernation and reproduction (*10*). As in New York, “bat in bedroom” was the most common exposure in Minnesota and 1 of the more frequent exposures in Colorado and Quebec.

In conclusion, during PEP guideline revision, which expanded the recommendation for PEP beyond persons with known bite exposures, numbers of bats submitted for testing, reported exposure incidents, and instances of PEP administration increased significantly in New York. Although the cause of the increases cannot be definitively determined, the increases were consistent with changes in guidelines and public education. With 89% of bats confirmed as rabies negative that were submitted because of possible human contact, improving bat capture and testing should be considered as a strategy for excluding rabies exposures and thus reducing the number of PEPs administered.

## References

[1] Eidson M, Bingman AK. Terrestrial rabies and human postexposure prophylaxis, New York, USA. Emerg Infect Dis. 2010;16:527–9. 10.3201/eid1603.090298 20202438 PMC3322005

[2] Human rabies prevention—United States, 1999. Recommendations of the Advisory Committee on Immunization Practices (ACIP). MMWR Morb Mortal Wkly Rep. 1999;48(RR-1):1–21. 10077411

[3] Eidson M, Schmit K, Keegan M, Trimarchi CV, Tserenpuntsag B, Willsey A. Development and evaluation of bat rabies education materials. Evidence-based. Prev Med. 2004;1:85–91.

[4] Chang HG, Eidson M, Noonan-Toly C, Trimarchi CV, Rudd R, Wallace BJ, Public health impact of reemergence of rabies, New York. Emerg Infect Dis. 2002;8:909–13. 12194765 10.3201/eid0809.010524 PMC2732541

[5] Wang X, DeMaria A, Smoke S, Brown CM, Han L. Bat rabies in Massachusetts, USA, 1985–2009. Emerg Infect Dis. 2010;16:1285–8. 10.3201/eid1608.100205 20678326 PMC3298317

[6] O’Bell SA, McQuiston J, Bell LJ, Ferguson SC, Williams LA. Human rabies exposures and postexposure prophylaxis in South Carolina, 1993–2002. Public Health Rep. 2006;121:197–202. 16528954 10.1177/003335490612100215 PMC1525274

[7] Pape WJ, Fitzsimmons TD, Hoffman RE. Risk for rabies transmission from encounters with bats, Colorado, 1977–1996. Emerg Infect Dis. 1999;5:433–7. 10.3201/eid0503.990315 10341181 PMC2640787

[8] Liesener AL, Smith KE, Davis RD, Bender JB, Danila RN, Neitzel DF, Circumstances of bat encounters and knowledge of rabies among Minnesota residents submitting bats for rabies testing. Vector Borne Zoonotic Dis. 2006;6:208–15. 10.1089/vbz.2006.6.208 16796518

[9] Huot C, De Serres G, Duval B, Maranda-Aubut R, Ouakki M, Skowronski DM. The cost of preventing rabies at any cost: post-exposure prophylaxis for occult bat contact. Vaccine. 2008;26:4446–50. 10.1016/j.vaccine.2008.06.076 18602958

[10] Frantz SC, Laniewicz BR. Comprehensive management of commensal bats. In: Brittingham MC, Kays J, McPeake R, editors. The ninth wildlife damage management conference proceedings. Lincoln (NE): Digital Commons@University of Nebraska—Lincoln; 2000. p. 172–86 [cited 2011 May 2]. http://digitalcommons.unl.edu/icwdm_wdmconfproc/27

